# Dietary diversity and iron deficiency anemia among a cohort of singleton pregnancies: a cross-sectional study

**DOI:** 10.1186/s12889-024-19294-z

**Published:** 2024-07-10

**Authors:** Nahla Al-Bayyari, Haleama Al Sabbah, Marah Hailat, Hadeel AlDahoun, Haya Abu-Samra

**Affiliations:** 1 Department of Nutrition and Food Processing, Faculty of Al-Huson University College, Al-Balqa Applied University, Al-Salt, Jordan; 2 Department of Nutritional Sciences, School of Public Health, University of Michigan, Ann Arbor, MI USA; 3 Department of Public Health, College of Health Sciences, Abu Dhabi University, Abu Dhabi, United Arab Emirates; 4 Yarmouk University, Irbid, Jordan; 5 American University School of the Middle East, Irbid, Jordan; 6 Abdullah Telfah Orthopedic Clinic, Irbid, Jordan

**Keywords:** Pregnancy, Iron deficiency, Anemia, Dietary diversity, Ferritin, Hemoglobin

## Abstract

**Background:**

Iron deficiency anemia (IDA) is considered one of the most common medical disorders observed during pregnancy. In low- and middle-income countries (LMICs), anemia and micronutrients deficiencies among pregnant women are associated with low consumption of animal products, monotonous starchy-diets, and seasonal consumption of vegetables and fruits.

**Methods:**

A cross-sectional study was conducted with 198 pregnant mothers aged between 19–45 years who visited the antenatal care clinics in Northern Jordan to document the prevalence of IDA and to describe the associations between dietary diversity, diet quality scores and oral iron supplementation with the pregnant women iron status. Participants were stratified into three groups by gestational age (*n* = 66 women per group). Gestational age, blood parameters, minimum dietary diversity score (MDD-W), and prime diet quality score for healthy (PDQSHF) and unhealthy foods (PDQSUF) were assessed using 24- hour dietary recall.

**Results:**

Prevalence of mild to moderate anemia was 27.8% among pregnant women. Third-trimester pregnant women were most affected. 52.5% have depleted iron stores (ferritin < 15 ng/ml), of them 30.8% have iron deficiency, and 21.7% have IDA. The (M ± SD) of the MDD-W, PDQSHF, and PDQSUF were 4.8 ± 1.6, 12.8 ± 3.9, and 7.2 ± 2.8 respectively. 52.5% achieved the MDD-W, 68% consumed < 4 servings/week of healthy food groups, and 50% consumed > 4 servings/week of unhealthy food groups. Mothers with higher MDD-W and PDQS had higher Hb and serum concentrations. Those taking iron supplements had significantly (*p* = 0.001) higher means of Hb, serum ferritin, and gestational weight gain. Significant differences were also found between PDQSHF, PDQSUF and the first and third trimester.

**Conclusions:**

Mild to moderate IDA is prevalent among pregnant mothers, especially in the third trimester. However, the prevalence of IDA among Jordanian pregnant women is lower than the global average. A high-quality, diverse diet, combined with oral iron supplementation and food fortification with iron, will help improve iron status, prevent anemia, and reduce its prevalence.

## Background

Anemia in general and iron deficiency anemia (IDA) in specific is still one of the global health problems in both developed and developing countries [1–3]. It is considered one of the most common medical disorders observed during pregnancy [4]. According to the World Health Organization (WHO), the global prevalence of anemia among pregnant mothers was around 38%, and 40% for iron deficiency. Iron deficiency (ID) is estimated to account for 50% of all cases of anemia in pregnancy [1, 5]. In Jordan, based on a national population household study conducted in 2017, the prevalence rate of anemia among pregnant mothers was 27.4%, of which; 65.2% have mild anemia [6].

Serum ferritin (SF) is a reliable biochemical marker of body iron storage, and its concentration in pregnancy is usually a good indicator of ID [7]. Iron requirements increase during pregnancy as gestational weeks increase to deliver oxygen and enzymes involved in fetal organogenesis [8]. In early pregnancy, anemia (including IDA); is associated with increased risks of gestational diabetes [9], preterm delivery, and low birth weight [10, 11]. Although low Hb is widely used as a valid diagnostic marker for anemia, it cannot reveal the stages of ID [8]. Consequently, the combination of Hb and SF reflects the spectrum of iron status perfectly more than Hb alone [9].

One of the dietary advice provided for pregnant mothers during antenatal visits is to increase their intake from various food groups to ensure quality and adequacy. Dietary diversification is considered an important approach to address micronutrient and macronutrient deficiencies. Indicators of dietary diversity which can be measured by identifying the number of food groups or different foods consumed over a specific reference period [12] are growingly used to measure the proxy for nutrient adequacy and diet quality in pregnancy [13]. However, consuming diverse food sources may ensure micronutrient adequacy; the choice of foods is pertinent to maternal diet quality and its impact on birth outcomes [14].

The elevated prevalence of anemia among pregnant mothers is attributable to a wide range of nutritional deficiencies such as iron, folic acid, vitamin B_12_ and B_6_. Socioeconomic status, low dietary diversity, low dietary quantity, gestational age, and iron supplement intake during pregnancy are identified as risk factors for anemia [15, 16]. Recently, a study in Abidjan found that the second and the third trimester of pregnancy, multipara, inadequate intake of energy, protein, and vitamin C and low dietary diversity are the significant and the independent determinants of IDA [17]. In low- and middle-income countries (LMICs), anemia and inadequate nutrients intake among pregnant women are associated with low consumption of animal products, monotonous starchy-diets, and seasonal consumption of vegetables and fruits [18]. Moreover, their consumption of foods known to reduce maternal anemia, such as foods rich in iron and vitamin A is low [19, 20]. During antenatal care, each pregnant mother is educated on good nutrition practices and provided with iron and folic acid supplements and are also encouraged to consume diverse food groups to meet the recommended dietary allowances during pregnancy [21]. However, other circumstances may hinder the early antenatal care onset, iron supplements intake and may restrict the affordability of an iron rich diet [22].

Even though numerous studies have been conducted to identify the prevalence of anemia among pregnant women in Jordan [6, 23], there are relatively few studies that address the effect of dietary intake on anemia [24], and none of them studied the effect of dietary diversity, the prime diet quality scores and iron supplements intake on the pregnant women’s iron status. Moreover, anemia among pregnant mothers, especially IDA requires comprehensive efforts to achieve further progress in the WHO goal regarding the reduction of IDA prevalence [25].

Despite these challenges, the Jordan Ministry of Health, along with other institutions, is working to achieve this goal through various programs. For instance, pregnant mothers after 13 weeks of gestation are supplemented with iron pills free of charge, and the Jordanian government has fortified flour with iron for the general population. During antenatal care visits, pregnant mothers are also counselled to focus on their dietary intake to improve pregnancy outcomes and to restore the nutritional status of mothers.

The objective of this study was to document the prevalence of iron deficiency anemia among pregnant mothers in Northern Jordan and to describe the associations between dietary diversity, diet quality scores and oral iron supplementation with the pregnant mothers’ iron status. Therefore, we hypothesized that pregnant women who are not taking iron supplements, who have a poor minimum dietary diversity, and low diet quality scores are at a greater risk of developing iron deficiency anemia.

## Methods

### Study design and recruitment

A cross-sectional study was conducted between August and December 2022 on pregnant mothers attending the Ministry of Health (MOH) antenatal care clinics located in maternal and child health centers in Northern Jordan.

Jordanian healthy mothers aged between 19–45 years old, who attended the MOH antenatal care clinics in Northern Jordan and pregnant with a singleton pregnancy (66 women in each pregnancy trimester) were included in this study. Whereas pregnant mothers diagnosed with pre-eclampsia, gestational diabetes, autoimmune disorders, chronic diseases (such as diabetes mellitus, liver, and renal diseases), hyperemesis gravidarum, and women with unknown pre-pregnancy weight (weight of the mother at conception or two weeks prior to conception or through the first two weeks of gestation) were excluded from this study.

### Sampling

“The sample size was calculated using the infinite population equation *n* = z^2^pq/d^2^. Where n stands for sample size, z is the value of the 95% confidence level, P is the estimated average prevalence, q is 1-p, and d is the accepted error which is the precision around the population mean” [26]. The prevalence of low-birth-weight newborns (13.8%) in Jordan according to Islam et al., 2020 [27] was used. Thus, the sample size required was:

$$\mathrm n={(1.96)}^2(0.138)\;(0.862)/\;{(0.05)}^2=182.8\;\mathrm{women}$$

The sample size was increased by at least 10% to increase the power of analysis and to compensate for excluded subjects from data analysis. Therefore, the total number included in the study was 198.

The 198 pregnant women were randomly selected from MOH antenatal care clinics located in the North of Jordan. Initially, all antenatal care clinics were numbered and entered into SPSS, from which six clinics were randomly selected using the SPSS computer random number generator. The study participants were also randomly selected based on their order number, with those having odd numbers being chosen. The selected pregnant women were divided into three groups based on gestational weeks (GWs): 1st trimester (0–13 weeks), 2nd trimester (14–26 weeks), and 3rd trimester (27–40 weeks), with 66 women in each group. The total number (*n* = 198) was distributed equally across the six antenatal clinics, with 33 women from each clinic and 11 from each trimester being randomly selected.

### Sociodemographic, medical, and antenatal care data

Data was collected directly from pregnant women through personal interviews using a validated questionnaire. Sociodemographic data include the mother's current age, marriage age, education and employment status of the mother and the husband, family income per month, religion, number of family members, health insurance, place of residence, and type of housing. The following medical data were included: last menstrual period, gestational age, history of chronic diseases, medications, gestational hypertension, gestational diabetes, preeclampsia/eclampsia, miscarriages, stillbirths, low birth weight, parity, spacing, previous deliveries, history of breastfeeding, and food allergies and intolerance. Additionally, information about antenatal care, such as the time of the first visit, the number of visits, the types of assessments, nutrition education, iron, and other micronutrient supplementations, were collected.

We specifically investigated the mother's current intake of iron supplements, emphasizing details regarding her supplementation routine, whether these supplements were prescribed during her prenatal and or antenatal care, any symptoms experienced due to iron intake, and whether they are taken in conjunction with other supplements. Additionally, we sought information on the frequency of supplement intake and the level of adherence to the recommended regimen. Therefore, the iron supplements intake was categorized into yes, if the pregnant woman started taking at least 30 mg of iron daily after the 13th week of gestation and was complying with the iron supplementation program and/or if the mother was taking iron supplements before pregnancy and continued during pregnancy according to her physician's prescribed dose. No, if the pregnant mother did not take any iron supplements on a daily basis after the 13th week of gestation.

#### Gestational age determination

Gestational age was calculated based on the date of the last menstrual period. For pregnant mothers who were not able to remember their last menstrual period date and/or were breastfeeding when they conceived, the gestational age was determined by the obstetrician, using ultrasonic fetal biometrics such as the biparietal diameter, abdominal circumference, and femur length.

### Anthropometric data

A stadiometer was used to measure height. Women were barefoot, minimally clothed and were asked to straighten their legs, adhere their heels, put their arms to the side, relax their shoulders and keep their heads in the Frankfort horizontal plane [28]. A beam scale was used to measure actual weight after its calibration and zero-balance check in each measurement. Women would stand without assistance on the scale’s center while being minimally dressed, barefoot and looking straight ahead [28]. Quetelet’s formula [weight (kg)/height (m)^2^] was used to calculate body mass index BMI [28]. Additionally, the mother’s pre-pregnancy weight was recorded from the mother's antenatal records. The gestational weight gain was calculated as the variation between the actual weight and the pre-pregnancy weight. Additionally, we considered differences in gestational weight gain based on pre-pregnancy BMI, following the recommendations of the Institute of Medicine. During the first trimester, weight gain may range from 1 to 5 pounds, or remain unchanged. In the second and third trimesters, mothers with a healthy pre-pregnancy weight typically gain between half a pound and 1 pound per week [29].

### Biochemical data

A venous blood sample was collected from the participants by the certified laboratory technician and distributed into two tubes: EDTA tube for complete blood count (CBC) analysis and a plain serum separator tube to determine the serum ferritin level. Blood samples were kept refrigerated and then sent to a certified diagnostic laboratory for analysis. To test for external validity of the biochemical blood analysis, two duplicated samples were sent to two different diagnostic laboratories to see the differences in the results. On the other hand, the same blood sample was divided into two tubes, each taking a different code and analysis was performed twice to test for internal validity. All blood analysis was carried out using two levels of different quality samples at the same laboratory and by the same team of laboratory technicians. Hematological parameters including complete blood count (CBC) of white blood cells (WBCs), red blood cells (RBCs), platelets, hemoglobin (Hb), hematocrit or packed cell volume (PCV), and RBCs indices of mean corpuscular volume (MCV), mean corpuscular hemoglobin (MCH), mean corpuscular hemoglobin concentration (MCHC), and red cell distribution width (RDW) were determined using MINDRAY (BC-5300) Auto Hematology Analyzer. All Hb concentrations were not adjusted for altitude at sea level because the participants live in areas with altitudes below 1000 m. Serum ferritin was measured by immunoassay using COBAS e 411 analyzer, (Roche Diagnostics, Rotkreuz, Switzerland).

### Dietary assessment

A trained dietitian was responsible for collecting the dietary intake from the participating mothers using 24-h recall and a validated quantitative food frequency questionnaire (FFQ) for Jordanian pregnant mothers [30]. Dietary intake was assessed using the minimum dietary diversity score for women (MDD-W) and the prime diet quality score (PDQS).

Each mother was asked to list the foods and the method of preparation, the amount eaten, and the time and place of food intake in the past 24 h. Food models, measuring cups, and spoons were utilized to help the participants estimate the portion sizes of the food and beverages they consumed. The MDD‐W was originally developed by the Food Agriculture Organization (FAO) in 2021 [31] as a population‐level dichotomous indicator to assess the sufficiency of micronutrients for reproductive-age women living in resource‐limited environments. The previous day's consumption of at least five out of ten food groups is defined as the MDD-W indicator. The ten food groups include starchy staples, peas and beans, nuts and seeds, dairy (milk and milk products), flesh foods (meat, fish, poultry), eggs, dark green vegetables rich in vitamin A, other fruits and vegetables rich in vitamin A, other vegetables, and other fruits. Based on the 24-dietary recall data, each of the consumed food groups receive 1 point, and the summation of the total points is identified as the MDD-W out of ten. The cumulative dietary diversity score was categorized into two outcome variables: dietary diversity (consuming > 5 food groups) and no dietary diversity (consuming < 5 food groups).

The PDQS is mainly composed of fourteen healthy food groups (dark green vegetables, carrots, cruciferous vegetables, other vegetables, whole citrus fruits, other fruits, whole grains, nuts and seeds, legumes, low‐fat dairy, eggs, fish, poultry, and liquid vegetable oils) and seven unhealthy food groups which include (red meat, processed meat, potatoes, refined grains, and baked goods, fried foods eaten away from home, sugar‐sweetened beverages, and ice cream and desserts). Other fruits and vegetables rich in vitamin A such as pumpkin, passion fruit, apricots, and mango were also included in the carrots group [32]. Based on the main component of mixed dish, it was either assigned to the healthy or unhealthy PDQS food group. The total number of weekly servings from each food item was calculated by the summation of the daily servings consumed from each food item included in each food group and then multiplied by seven. Pregnant mothers who consumed food items were grouped into either healthy or unhealthy PDQS food groups. Based on the total food serving(s) consumed per week from both the healthy and unhealthy food groups, a score for each food group was allocated as the following: healthy food groups: 2 points for 4 + serving/week, 1 point for 2–3 serving/week, and 0 points for 0–1 serving/week. Unhealthy food groups: 0 points for 4 + serving/week, 1 point for 2–3 serving/week, and 2 points for 0–1 serving/week. The sum of the scores for each food group was used to get the overall PDQS score.

### Statistical analysis

The collected data were double entered, checked, and analyzed using SPSS statistical package (IBM, SPSS version 25, 2017). Descriptive statistics were performed using frequencies and percentages to describe the categorical variables. Means and standard deviations (SD) were used to describe continuous variables. The nonparametric Kolmogorov–Smirnov test was performed to examine all continuous variables for normal distribution. A Student t-test for independent variables was performed to detect any significant differences between the means of the normally distributed continuous variables. The numeric variables of gestational age, Hb, serum ferritin, MDD-W, and PDQS were converted into categorical variables according to international and/or laboratory cutoff values. The associations between dichotomous and categorical variables were assessed using Pearson’s Chi-square (χ2) and Fisher’s exact tests.

## Results

### Demographic and anthropometric data

A total of 458 mothers were screened for eligibility, out of which 260 did not meet the inclusion criteria and were excluded from the study. The remaining 198 pregnant mothers met the inclusion criteria and were included in the study analysis (Supporting information).

The mean and standard deviation (M ± SD) of the study participants' age was (29 ± 5.9) years, height (160.5 ± 5.7) cm, and pre-pregnancy weight was (65.9 ± 14.0) kg. All the mothers were Muslims, 98.5% married and living with their husbands, 55.6% resided in rural areas, 25% lived in rented houses, the majority (90%) were unemployed, 26% of them had a family monthly income below 300 JD, 94% have health insurance, about half (48%) of them were highly educated, and 48% were overweight or obese before pregnancy (Table 1).

**Table 1:** Demographic and anthropometric characteristics of the study participants (*N* = 198)

**Variable**	**n**	**%**
**Religion**		
-Muslim	198	100
**Social** **status**		
-Married and living with husband	195	98.5
-Married and not living with husband	3	1.5
**Area of residence**		
-Urban	88	44.4
-Rural	110	55.6
**Housing**		
-Owned	148	74.7
-Rent	50	25.3
**Mother employment**		
-Employed	19	9.6
-Not employed	179	90.4
**Father employment**		
-Employed	181	91.4
-Not employed	17	8.6
**Family monthly income (JD)**		
-< 300	51	25.7
-301-500	132	66.7
-> 500	15	7.6
**Health insurance**		
-Ensured	187	94.4
-Not ensured	11	5.6
**Mother level of education**		
-Elementary	30	15.2
-Secondary	74	37.4
-Diploma	23	11.6
-University	71	35.9
**Body mass index prior to pregnancy (kg/m **^**2**^)		
-Underweight (< 18.5)	9	4.5
-Normal weight (18.5-24.99)	94	47.5
-Overweight (25-29.99)	63	31.8
-Obese (> 30)	32	16.2
**Mean ± SD**		
** Age (years)**	29+5.9	
** Height (cm)**	160.5+5.7	
** Pre-pregnancy weight (kg)**	65.9+14.0	
** Gestational weight gain (kg)**	5.3+5.7	

Data are presented as frequencies (n) and percentages (%) unless otherwise stated

### Iron supplementation and anemia

Of the total participating pregnant mothers in the second and third trimesters (*n* = 132), about two-thirds (69%) of them took iron supplements after 13 weeks of gestation, 45% took iron supplements directly after the meal, and 95% took iron supplements with water. In addition, 47% took other supplements such as zinc and calcium with iron, and 40% suffered from constipation as a side effect of iron supplements. Among the 198 mothers, 28% have a Hb level below 11 g/dl. None of them have severe anemia (Hb < 7 g/dl), 15% have mild (10–10.9 g/dl) anemia, and around 13% have moderate (7–9.9 g/dl) anemia. The prevalence of anemia in the third trimester was the highest (36%), followed by the second trimester (23%), and the lowest was found among the first trimester (11%) pregnant mothers (Table 2).

**Table 2:** Characteristics of iron supplementation practices and anemia among study participants

**Variable**	**n**	**%**
**Iron supplements after 13 weeks of gestation (*n*** = 132)		
-Yes	91	68.9
-No	41	31.1
**Iron supplements intake (*n*** = 91)		
-2 hours after meal	34	37.4
-2 hours before meals	5	5.5
-With meal	3	3.3
-Directly after meal	41	45.0
-On empty stomach	8	8.8
**Iron supplements intake with (*n*** = 91)		
-Water	86	94.5
-Juice	5	5.5
**Taking iron with other supplements (as Zinc and calcium) (*n*** = 91)		
-Yes	43	47.3
-No	48	52.7
**Symptoms with iron supplements intake (*n*** = 91)		
-Nausea	15	16.4
-Cramps	3	3.3
-Gases	2	2.2
-Constipation	36	39.6
-No symptoms	35	38.5
**Anemia (*n* = 198)**		
-Non anemic (Hb > 11 g/dl)	143	72.2
-Anemic (Hb < 11 g/dl)	55	27.8
**Anemia category (*n* = 198)**		
-Non anemic (Hb > 11 g/dl)	143	72.2
-Mild (Hb 10-10.9 g/dl)	30	15.2
-Moderate (Hb 7.0-9.9)	25	12.6
-Severe (Hb < 7.0 g/dl)	0	0.0
**Anemia in each pregnancy trimester (*n* = 66)**		
-First (Hb < 11 g/dl)	7	10.6
-Second (Hb < 10.5 g/dl)	15	22.7
-Third (Hb < 11 g/dl)	24	36.4

Data are presented as frequencies (n) and percentages (%)

**Fig. 1:**
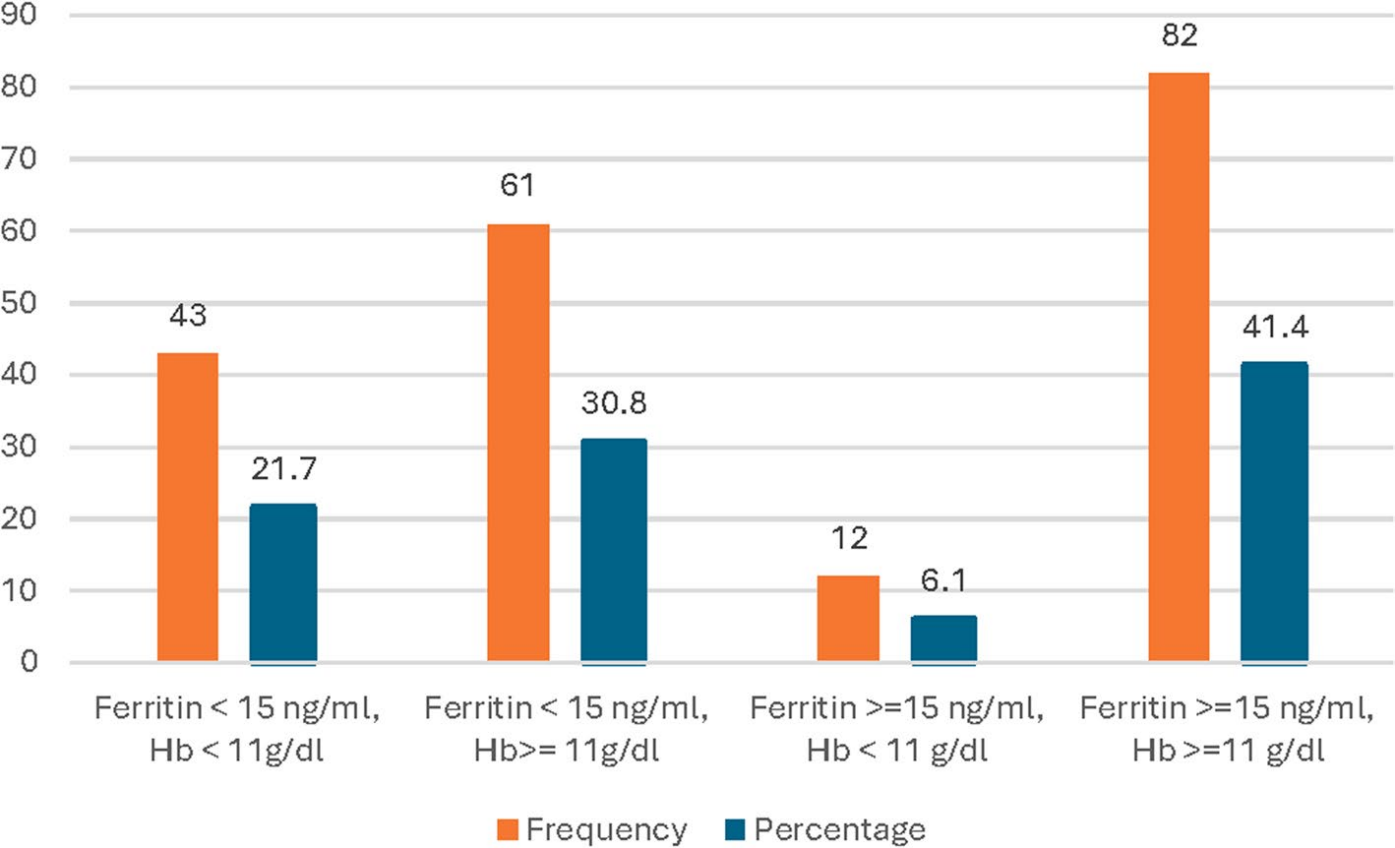
Iron status of study participants according to ferritin and hemoglobin levels (*N* = 198)

Figure 1 shows the iron status of all participating pregnant mothers (*N* = 198) according to their serum ferritin and Hb levels. Around 41.5% of them had normal (serum ferritin ≥ 15 ng/ml, Hb ≥ 11 g/dl) iron status. 52.5% had depleted iron stores, of them 21.7% had iron deficiency anemia (serum ferritin < 15 ng/ml, Hb < 11 g/dl), and 30.8% had iron deficiency without anemia (serum ferritin < 15 ng/ml, Hb ≥ 11 g/dl). 6.1% had anemia without iron deficiency (serum ferritin ≥ 15 ng/ml, Hb < 11 g/dl).

Mean comparison of blood parameters according to iron supplements intake using students' independent t-test showed that there are statistically significant differences in gestational weight gain, RBCs, Hb, MCHC, platelets and serum ferritin between pregnant mothers taking iron supplements and those not taking iron supplements. Those taking iron supplements had significantly higher means of gestational weight gain, RBC, Hb, MCHC, platelets, and serum ferritin (Table 3).

**Table 3:** Mean differences of gestational weight gain, dietary diversity, and blood parameters according to iron supplements intake (*N* = 198)

**Variable**	**Iron ** **supplements** **intake**		***P*-Value**
	**Yes** ***(n* = 91)** **Mean ± SD**	**No** **(*n* = 107)** **Mean ± SD**	
Gestational weight gain (kg)	7.8+6.0	3.2+4.5	0.001
RBCs (´10^Ù^12/L)	4.3+0.4	4.0+0.4	0.001
PCV (%)	37.9+33.5	37.0+3.3	0.790
Hb (g/dl)	11.9+1.2	11.2+1.2	0.001
MCV (fL)	87.4+7.7	86.7+6.7	0.513
MCH (pg)	28.0+3.1	28.0+2.6	0.957
MCHC (g/dl)	32.3+0.8	31.9+1.4	0.036
Platelets (´10^Ù^9/L)	231.7+60.4	207.2+61.2	0.005
WBCs (´10^Ù^9/L)	18.0+88.5	8.0+1.9	0.245
RDW (%)	13.9+1.6	14.4+11.2	0.671
Serum ferritin (ng/ml)	27.4+25.8	17.0+12.4	0.001
MDD-W	4.7+1.6	4.8+1.5	0.557
PDQSHF	12.6+3.9	12.9+3.8	0.674
PDQSUF	6.9+2.7	7.4+2.9	0.180

Data are presented as means + standard deviations

*P*-value < 0.05 was considered significant for student independent t-test

*RBCs* Red Blood Cells, *PCV* Packed Cell Volume, *Hb* Hemoglobin, *MCV* Mean Corpuscular Volume, *MCH* Mean Corpuscular Hemoglobin, *MCHC* Mean Corpuscular Hemoglobin Concentration, *WBCs* White Blood cells, *RDW* Red Cell Distribution Width, *MDD-W* Minimum Dietary Diversity for Women, *PDQSHF* Prime Diet Quality Score for Healthy Foods, *PDQSUF* Prime Diet Quality Score for Unhealthy Foods

### Dietary diversity

The dietary analysis of the MDD-W based on the 24-h recall of pregnant mothers showed that 96% consume starchy grains, while milk and milk products, meats, and other fruits not rich in vitamin A were consumed by 71%, 73%, and 64%, respectively. Nevertheless, 66%, 86%, 67%, 80%, 76%, and 53% did not consume any legumes, nuts and seeds, eggs, dark green vegetables rich in vitamin A, other fruits, or vegetables rich in vitamin A and other vegetables, respectively. The overall mean and standard deviation (M ± SD) score of the food groups consumed was (4.8 ± 1.6) which is around the MDD-W (Table 4).

**Table 4:** Minimum dietary diversity scores (MDD-W) of food groups consumed by pregnant mothers (*N* = 198)

**Food group**	**Yes** **(1 point)** **n (%)**	**No** **(0 Point)** **n (%)**	
Starchy grains	190 (96.0)	8 (4.0)	
Legumes (Beas, beans, …. etc.)	67 (33.8)	131 (66.2)	
Nuts and seeds	27 (13.6)	171 (86.4)	
Milk and milk products	141 (71.2)	57 (28.8)	
Meats (Fish, poultry, red meat)	145 (73.2)	53 (26.8)	
Eggs	65 (32.8)	133 (67.2)	
Dark green vegetables rich in vitamin A	39 (19.7)	159 (80.3)	
Other vegetables and fruits rich in vitamin A	48 (24.2)	150 (75.8)	
Other vegetables (Okra, cabbage, cauliflower, …etc.)	93 (47.0)	105 (53.0)	
Other fruits (Grapes, pomegranate, figs, citrus, ... etc.)	126 (63.6)	72 (36.4)	
MDD-W	**Minimum score**	**Maximum score**	**Mean ± SD**
	1.0	9.0	4.8+1.6

Data are presented as frequencies (n) and percentages (%) unless otherwise stated

*MDD-W* Minimum Dietary Diversity for Women

The PDQS of the healthy and unhealthy food groups are presented in Table 5. Results showed that the majority of pregnant mothers consumed low amounts (0–1 serving/week) of several healthy food items, such as dark green vegetables, legumes, fish, and low-fat dairy products. Conversely, high consumption rates (> 4 servings/week) were noted for other vegetables, fruits, poultry, and vegetable oils. The overall mean score for healthy food group consumption (PDQSHF) was 12.8 ± 3.9.

**Table 5:** Prime diet quality scores (PDQS) of healthy and unhealthy food groups consumed by pregnant mothers (*N* = 198)

**Healthy food groups**	**0-1** **servings/week** **(0 Points)** **n (%)**	**2-3 servings/week** **(1 point)** **n (%)**	**≥ 4 servings/week** **(2 points)** **n (%)**
Dark green vegetables	102 (51.5)	52 (26.3)	44 (22.2)
Cruciferous vegetables	151 (76.3)	34 (17.2)	13 (6.6)
Carrot	150 (75.8)	29 (14.6)	19 (9.6)
Other vegetables	16 (8.1)	29 (14.6)	153 (77.3)
Citrus (Whole fruit)	56 (28.3)	46 (23.2)	96 (48.5)
Other fruits	60 (30.3)	29 (14.6)	109 (55.1)
Legumes	113 (57.1)	50 (25.3)	35 (17.7)
Nuts and seeds	104 (52.5)	34 (17.2)	60 (30.3)
Poultry	25 (12.6)	12 (6.1)	161 (81.3)
Fish	117 (59.1)	59 (29.8)	22 (11.1)
Eggs	82 (41.4)	38 (19.2)	78 (39.4)
Whole grains	106 (53.6)	45 (22.7)	47 (23.7)
Vegetable oils	2 (1.0)	6 (3.0)	190 (96.0)
Low fat milk and milk products	191 (96.5)	2 (1.0)	5 (2.5)
**PDQSHF**	**Minimum score**	**Maximum score**	**Mean ± SD**
	4.0	22.0	12.8 +3.9
**Unhealthy food groups**	**≥ 4 servings/week** **(0 points)** **n (%)**	**2-3 servings/week** **(1 point)** **n (%)**	**0-1 servings/week** **(2 points)** **n (%)**
Potato	110 (55.6)	46 (23.2)	42 (21.2)
Red meat	59 (29.8)	60 (30.3)	79 (39.9)
Processed meat	38 (19.2)	27 (13.6)	133 (67.2)
Refined grains and packed foods	73 (36.9)	14 (7.1)	111 (56.1)
Sweetened beverages	88 (44.4)	34 (17.2)	76 (38.4)
Fried foods from outside home	69 (34.8)	40 (20.2)	89 (44.9)
Ice creams and sweets	114 (57.6)	31 (15.7)	53 (26.8)
**PDQSUF**	**Minimum score**	**Maximum score**	**Mean ± SD**
	0.0	12.0	7.2+2.8

Data are presented as frequencies (n) and percentages (%) unless otherwise stated

*PDQSHF* Prime Diet Quality Score for Healthy Foods, *PDQSUF* Prime Diet Quality Score for Unhealthy Foods

For unhealthy food groups, more than half of the pregnant mothers consumed high amounts of potatoes, ice cream, and sweets (> 4 servings/week), while a significant portion had low consumption (0–1 serving/week) of red meat, processed meat, refined grains, sweetened beverages, and fried foods from outside the home. The overall mean score for unhealthy food group consumption (PDQSUF) was 7.2 ± 2.8 (Table 5).

The PDQS of the healthy and unhealthy food groups are presented in Table 5. Results showed that around 52%, 76%, 75.8%, 57%, 53%, 59%, 41%, 54%, and 97% of pregnant mothers consume (0–1 serving/week) of dark green vegetables, cruciferous vegetables, carrots, legumes, nuts and seeds, fish, eggs, whole grains, and low-fat milk and milk products, respectively. On the other hand, around 77%, 49%, 55%, 81%, and 96% consumed (> 4 serving/week) of other vegetables, whole citrus fruit, other fruits, poultry, and vegetable oils. The overall mean (SD) of the consumed healthy food groups score per week (PDQSHF) was 12.8 ± 3.9. Unhealthy food group consumption results revealed that more than 50% of pregnant mothers consume (> 4 servings/week) of potatoes, ice cream, and sweets. Nonetheless, 40%, 67%, 56%, 38%, and 50% consume (0–1 serving/week) of red meat, processed meat, refined grains, and packed foods, sweetened beverages, and fried foods from outside the home, respectively. The overall mean and standard deviation (M ± SD) of the weekly consumption of unhealthy food groups score (PDQSUF) was 7.2 ± 2.8 (Table 5).

### Dietary diversity, blood parameters, gestational age, and *iron* supplements intake

Table 6 shows that around 48% of the pregnant mothers did not achieve the MDD-W, 68% their PDQSHF was below or equal to 14 and 50% had a PDQSUF above 7. No statistically significant differences were found between the first, second, and third trimesters and the MDD-W, PDQSHF and PDQSUF. Although, significant differences were found between the first and third trimesters and the PDQSHF, and the PDQSUF, where pregnant mothers in the third trimester had a significantly higher proportion of PDQSHF below or equal to 14 and a lower proportion of PDQSUF above 7. Moreover, the student independent t-test revealed statistically no significant differences between the MDD-W, PDQSHF and PDQSUF, and the iron supplements intake (Table 3).

**Table 6:** Dietary diversity scores, hemoglobin, and serum ferritin levels of pregnant mothers according to gestational age (*N* = 198)

**Variable**	**Total** **(*n* = 198)** **n (%)**	**First trimester** **(*n* = 66)** **n (%)**	**Second trimester** **(*n* = 66)** **n (%)**	**Third trimester** **(*n* = 66)** **n (%)**	***P*-Value**
**MDD-W**					
-< 5	94 (47.5)	31 (47.0)	33 (50.0)	30 (45.5)	0.868
-≥ 5	104 (52.5)	35 (53.0)	33 (50.0)	36 (54.5)	
**PDQSHF**					
-≤ 14	134 (67.7)	40 (60.6)^c^	42 (63.6)	52 (78.8)^c^	0.057
-> 14	64 (32.3)	26 (39.4)	24 (36.4)	14 (21.2)	
**PDQSUF**					
-≤ 7	100 (50.5)	29 (43.9)^c^	30 (45.5)	41 (62.1)^c^	0.068
-> 7	98 (49.5)	37 (56.1)	36 (54.5)	25 (37.9)	
**Hemoglobin (g/dl)**					
-< 11	55 (27.8)	7 (10.6)^a,c^	24 (36.4)^a^	24 (36.4)^c^	0.001
-≥ 11	143 (72.2)	59 (89.4)	42 (63.6)	42 (63.6)	
**Ferritin (ng/ml)**					
-< 15	104 (52.5)	21 (31.8)^a,c^	38 (57.6)^a^	45 (68.2)^c^	0.001
-≥ 15	94 (47.5)	45 (68.2)	28 (42.4)	21 (31.8)	

Data are presented as frequencies (n) and percentages (%)

*P*-value < 0.05 considered significant for Chi-Square test

*MDD-W* Minimum Dietary Diversity for Women, *PDQSHF* Prime Diet Quality Score for Healthy Food, *PDQSUF* Prime Diet Quality Score for Unhealthy Food

^a^Indicates a significant difference (*p* < 0.05) between first and second trimester using Chi-Square test

^c^Indicates a significant difference (*p* < 0.05) between the first and the third trimester using Chi-Square test

Statistically, significant (*p* = 0.001) differences were noticed between the first, second, and third trimesters and Hb and serum ferritin levels. Pregnant mothers in third trimester showed significantly higher proportions of Hb < 11 g/dl and serum ferritin < 15 ng/ml (Table 6). Despite this, between the MDD-W, PDQSHF, and PDQSUF and the Hb and serum ferritin, there were not any statically significant differences detected (Table 7).

**Table 7:** Dietary diversity scores according to pregnant mothers’ hemoglobin, and serum ferritin levels (*N* = 198)

**Variable**	**Total**	**Hemoglobin**		***P*-Value**	**Serum Ferritin**		***P*-Value**
		**< 11 g/dl** **(*n* = 55)** **n (%)**	**≥ 11 g/dl** **(*n* = 143)** **n (%)**		**< 15 ng/ml** **(*n* = 104)** **n (%)**	**≥ 15 ng/ml** **(*n* = 94)** **n (%)**	
**MDD-W**							
-< 5	94 (47.5)	25 (45.5)	69 (48.3)	0.724	44 (42.3)	50 (53.2)	0.126
-≥ 5	104 (52.5)	30 (54.5)	74 (51.7)		60 (57.7)	44 (46.8)	
**PDQSHF**							
-≤ 14	134 (67.7)	39 (70.9)	95 (66.4)	0.546	68 (65.4)	66 (70.2)	0.468
-> 14	64 (32.3)	16 (29.1)	48 (33.6)		36 (34.6)	28 (29.8)	
**PDQSUF**							
-≤ 7	100 (50.5)	30 (54.5)	70 (49.0)	0.481	52 (50.0)	48 (51.1)	0.881
-> 7	98 (49.5)	25 (45.5)	73 (51.0)		52 (50.0)	46 (48.9)	

Data are presented as frequencies (n) and percentages (%)

*P*-value < 0.05 considered significant for Chi-Square test

*MDD-W* Minimum Dietary Diversity for Women, *PDQSHF* Prime Diet Quality Score for Healthy Food, *PDQSUF* Prime Diet Quality Score for Unhealthy Food

**Fig. 2:**
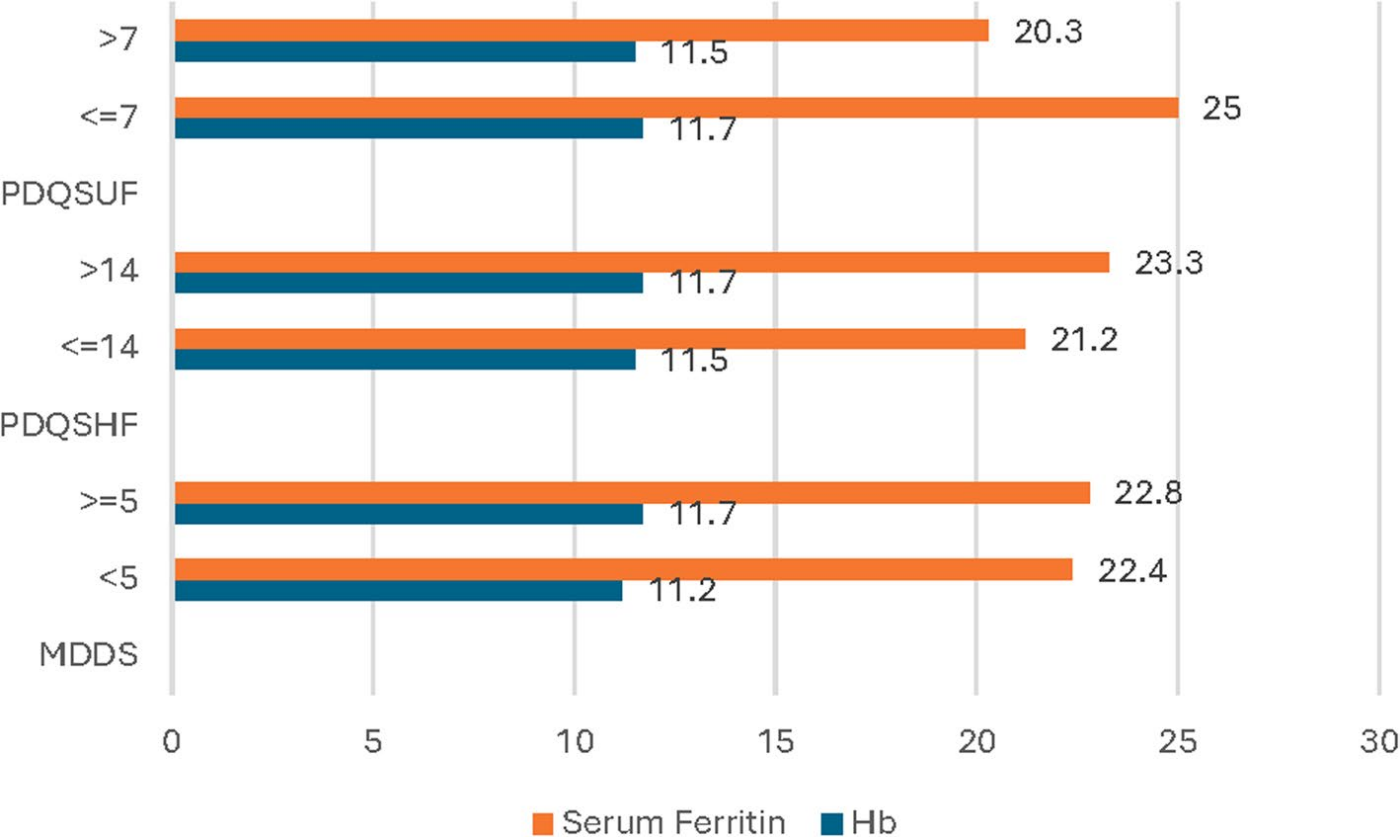
Means of the Hb and serum ferritin levels according to MDD-W, PDQSHF and PDQSUF (*N* = 198)

Finally, with one exception, we did not observe statistically significant differences in Hb or serum ferritin concentrations among women who did or did not consume foods from specific food groups. The exception was that the mean Hb concentration among women who consumed starchy grains was statistically significantly higher than among those that did not consume starchy grains. However, women eating different food groups in general have higher means of Hb and serum ferritin levels, although the differences do not reach the level of significance (Table 8). Additionally, the overall Hb and serum ferritin levels when studied with MDD-W showed higher means of Hb (11.7 ± 1.3) and serum ferritin (22.8 ± 23.0) levels among mothers who have higher diversity (MDD-W > 5) comparing to low diversity (MDD-W < 5) (11.2 ± 1.5) and (22.4 ± 19.5) respectively. Mothers who have PDQSHF > 14 and PDQSUF < 7 had also higher means of Hb and serum ferritin levels compared to mothers who have PDQSHF < 14 and PDQSUF > 7 although no significant differences were found (Fig. 2).

**Table 8:** Mean differences in Hemoglobin and serum ferritin levels according to dietary diversity (*N* = 198)

Food group	Hemoglobin level Mean ± SD		*P*-value	Serum ferritin Mean ± SD		*P*-value
	**Yes**	**No**		**Yes**	**No**	
Starchy grains	11.6 ± 1.2	10.8 ± 1.7	0.050	23.0 ± 21.6	14.1 ± 12.4	0.250
Legumes (Beas, beans, …. etc.)	11.7 ± 1.2	11.5 ± 1.3	0.557	25.9 ± 22.4	20.9 ± 20.7	0.120
Nuts and seeds	11.8 ± 1.3	11.6 ± 1.2	0.374	29.8 ± 27.0	21.5 ± 20.2	0.060
Milk and milk products	11.6 ± 1.3	11.5 ± 1.2	0.527	22.4 ± 20.0	23.2 ± 24.4	0.820
Meats (Fish, poultry, red meat)	11.7 ± 1.2	11.4 ± 1.3	0.169	25.1 ± 24.2	21.7 ± 20.2	0.325
Eggs	11.6 ± 1.2	11.5 ± 1.4	0.385	24.5 ± 21.5	18.8 ± 20.7	0.074
Dark green vegetables rich in vitamin A	11.8 ± 1.2	11.5 ± 1.3	0.319	25.1 ± 28.2	22.0 ± 19.3	0.421
Other vegetables and fruits rich in vitamin A	11.6 ± 1.2	11.5 ± 1.3	0.709	25.9 ± 27.3	21.6 ± 19.0	0.228
Other vegetables (Okra, cabbage, cauliflower, …etc.)	11.6 ± 1.2	11.6 ± 1.3	0.902	22.9 ± 20.0	22.5 ± 22.5	0.896
Other fruits (Grapes, pomegranate, figs, citrus, … etc.)	11.7 ± 1.3	11.4 ± 1.2	0.169	22.6 ± 22.2	22.8 ± 19.8	0.950

Data are presented as means ± standard deviations

*P*-value < 0.05 was considered significant for student independent t-test

## Discussion

This study assessed iron status and dietary diversity of pregnant mothers attending antenatal care clinics in Northern Jordan. In addition, it determined the impact of achieving the MDD-W and the prime-diet quality scores of healthy (PDQSHF); and unhealthy (PDQSUF) foods consumptions through the pregnancy trimesters on iron status. Results revealed that 27.8% of the study participants have mild to moderate anemia and 21.7% have IDA. Anemia was more prevalent (36.4%) among pregnant mothers in their third trimester. The WHO global prevalence of anemia is 30% among non-pregnant women and 38% among pregnant women [1]. In Jordan, the prevalence of anemia among non-pregnant women is 19.3%, and 27.4% among pregnant women [6]. Therefore, the anemia prevalence in Jordan is lower than the WHO global prevalence for both pregnant and non-pregnant women, indicating that Jordanian pregnant mothers might have better nutritional status and antenatal health care.

The prevalence of IDA in our study is lower than the 30.4% reported in the Abidjan study [17] and the previous Jordan national population household study conducted in 2017 [6]. However, it is higher compared to the prevalence of anemia (26.5%) among pregnant women in Northern Jordan and in the third trimester (32.0%) as reported in the last Jordan national micronutrient and nutrition survey for 2019 [33]. Despite these differences, there is agreement on the severity of anemia, as all cases in our study suffer from mild to moderate anemia [6, 33]. A recent screening study of IDA among pregnant women in Saudi Arabia found the overall prevalence of anemia (Hb < 11 mg/dl) to be 28.6%, with prevalence by trimester being 12.2% in the first, 37.2% in the second, and 36.3% in the third trimester [34]. These results are somewhat similar to our findings, particularly the prevalence of anemia in the third trimester (36.4%). However, we report a lower prevalence in the first (10.6%) and second trimesters (22.7%), as well as a lower overall anemia prevalence.

In line with the WHO report, we used the cutoff < 15 ng/ml for SF concentration as an indicator of depleted iron stores [5] which was found in more than half of our participants. Similar findings were observed in a recent multicenter study conducted on pregnant mothers in Latvia [35]. Low SF concentration is predictive of late anemia in the third trimester, and we found 68.2% of pregnant mothers in the third trimester have SF below 15ng/ml, and the mean SF concentration was 14.7 ± 10.7. In contrast, Muthukumaran et al. study [36] reported higher means (16.62 ± 73.42) and a lower proportion (60%) of low SF concentrations.

Our study found that around 53% of pregnant women achieve the MDD-W, which is close to the percentage (55%) reported by Shrestha et al. [37], although we had around 47% of the mothers who didn’t achieve the MDD-W. It also revealed that pregnant women in Northern Jordan consume a low score dietary diversity diet. The mean dietary diversity score (4.8 ± 1.6) of pregnant women in this study is like the mean MDD-W (4.76 ± 1.23) reported in the western hill region of Nepal [37], but it is lower compared to the MDD-W of pregnant women from Kenya [38], Bangladesh [39], Pakistan [40], and Cameroon [21], and higher compared to MDD-W of pregnant mothers from Ethiopia [41], and Malawi [42]. The variations in the MDD-W may be due to differences in geographical location, sociodemographic characteristics, cultural factors, sample size, and the time of the year the data was collected.

In the Mount Cameroon area, Jugha et al. [21] found that a daily intake of animal protein, dairy, and vitamin A-rich fruits and vegetables was associated with a significantly lower prevalence of anemia. They also reported that pregnant women with less diverse diets had lower mean Hb levels (10.85 ± 1.33) compared to those with more diverse diets (12.39 ± 1.34). Similarly, our study found that the mean Hb level of women with more dietary diversity was higher than those with lower dietary diversity. Additionally, we observed higher serum ferritin levels among women with more dietary diversity. However, unlike Jugha et al.'s findings, we did not find a significant difference in Hb levels related to the intake of dairy, animal protein, or vitamin A-rich fruits and vegetables. The exception was the consumption of starchy grains, where significantly higher Hb levels were found among those who consume starchy grains daily. This result is likely due to the Jordanian government's bread fortification program with iron [33].

Although dietary diversity is not the only contributing factor of anemia in pregnancy, Jugha and his colleagues [21] reported that the most pressing constraint in their study was dietary diversity, where a low dietary diversity score (< 5 food groups) increased the likelihood of anemia by around ten folds when compared with high dietary diversity score (≥ 5 food groups). In addition, they reported that dietary diversity positively affects Hb concentrations despite the gestational age, and more than 80% of reported cases of anemia were referred to dietary diversity [21]. Similar findings were reported by Lebso et al. [15] and Delil et al. [43] in Southern Ethiopia. In contrast, Saaka et al. in the Northern Ghana study, revealed that “diet was not one of the protective factors against anemia” [44]. Even though, our results did not show significant differences between dietary diversity scores and iron status, but it showed that women who have higher diversity scores have better levels of SF and Hb.

Oral iron supplementation is the first rule of treatment for pregnant women with iron-deficient anemia [45]. However, compliance with oral iron supplements administration during pregnancy is becoming a problem due to side effects such as constipation which may discourage women from taking the iron supplements [29]. In our study, 70% of the mothers took their iron supplements as prescribed, and around 62% reported that they suffered from side effects. Nevertheless, iron supplementation increases maternal hematopoiesis, erythropoiesis, and fetal growth more in the second pregnancy [46]. Additionally, a recent study reported that treatment of iron deficiency anemia by iron supplementation enhances appetite and lowers ghrelin levels [47]. This might explain the significantly higher mean in pregnancy weight gain among pregnant mothers taking iron supplements compared to mothers not taking iron supplements during pregnancy in our study.

In 2020 Agbozo et al. [48] reported significantly higher levels of Hb, RBCs, hematocrit, and MCV among pregnant mothers with adequate nutrient intake. In this study, mothers taking iron supplements during pregnancy had significantly higher means of gestational weight gain, RBCs, Hb, MCHC, and serum ferritin. The increased bioavailability of iron during pregnancy and the direct effect of iron supplementation on improving appetite and food intake may contribute to these outcomes.

Although our study has some strengths, such as measuring the mother's Hb, and serum ferritin as well as assessing the MDD-W along with PDQS, it has some limitations; the study design was cross-sectional, and it is relatively difficult to establish a causal relationship and to investigate the temporal relationship between outcomes and risk factors. The sample size was somehow small and might not be representative of all mothers living in the North of Jordan. Serum ferritin concentrations were not adjusted for inflammation. Additionally, we did not measure any inflammatory biomarkers such as C-reactive protein, nor did we measure the soluble serum transferrin receptor. Dietary diversity was determined using 24-h recall, and this method has limitations such as it relies on memory, underreporting/and or overreporting may occur, and it is seldom representative of a person’s usual dietary intake.

## Conclusions

Mild to moderate iron deficiency anemia (IDA) is still prevalent among Jordanian pregnant mothers, especially in the third trimester. However, the prevalence of IDA in this population is lower than the global average, indicating progress towards reducing anemia among reproductive-age women, particularly pregnant mothers. Differentiating between types of anemia before treatment and iron supplementation is highly recommended. Additionally, health and nutrition education are crucial for improving the nutritional status of pregnant mothers and decreasing the prevalence of anemia. A high-quality, diverse diet, along with oral iron supplementation and food fortification with iron, will support and potentially help in preventing anemia.

## Data Availability

Data is available through a request to corresponding author.

## Abbreviations

IDA: Iron Deficiency Anemia
WHO: World Health Organization
ID: Iron Deficiency
Hb: Hemoglobin
SF: Serum Ferritin
CDC: Centers for Disease Control
LMICs: Low- and middle-income countries
MOH: Ministry of Health
SPSS: Statistical Package for Social Sciences
GWs: Gestational Weeks
BMI: Body Mass Index
FFQ: Food Frequency Questionnaire
MDD-W: Minimum Dietary Diversity for Women
PDQS: Prime Diet Quality Score
PDQSHF: Prime Diet Quality Score for Healthy Food
PDQSUF: Prime Diet Quality Score for Unhealthy Food
CBC: Complete Blood Count
RBCs: Red Blood Cells
PCV: Packed Cell Volume
MCV: Mean Corpuscular Volume
MCH: Mean Corpuscular Hemoglobin
MCHC: Mean Corpuscular Hemoglobin Concentration
WBCs: White Blood cells
RDW: Red Cell Distribution Width
